# Three-Dimensional Metal-Insulator-Metal Decoupling Capacitors with Optimized ZrO_2_ ALD Properties for Improved Electrical and Reliability Parameters

**DOI:** 10.3390/ma15238325

**Published:** 2022-11-23

**Authors:** Konstantinos Efstathios Falidas, Kati Kühnel, Matthias Rudolph, Maximilian B. Everding, Malte Czernohorsky, Johannes Heitmann

**Affiliations:** 1 Fraunhofer Institute for Photonic Microsystems (IPMS), Center Nanoelectronic Technologies (CNT), An der Bartlake 5, 01109 Dresden, Germany; 2 Institute of Applied Physics, TU Bergakademie Freiberg, Leipziger Str. 23, 09599 Freiberg, Germany

**Keywords:** high-κ, dielectric, ZrO_2_, Al-doping, MIM capacitors, 3-D MIM capacitors, decoupling capacitors

## Abstract

Embedded three-dimensional (3-D) metal-insulator-metal (MIM) decoupling capacitors with high-κ dielectric films of high capacitance and long-life time are increasingly needed on integrated chips. Towards achieving better electrical performance, there is a need for investigation into the influence of the variation in atomic layer deposition (ALD) parameters used for thin high-κ dielectric films (10 nm) made of Al_2_O_3_-doped ZrO_2_. This variation should always be related to the structural uniformity, the electrical characteristics, and the electrical reliability of the capacitors. This paper discusses the influence of different Zr precursor pulse times per ALD cycle and deposition temperatures (283 °C/556 K and 303 °C/576 K) with respect to the capacitance density (C-V), voltage linearity and leakage current density (I-V). Moreover, the dielectric breakdown and TDDB characteristics are evaluated under a wide range of temperatures (223–423 K).

## 1. Introduction

Placing a relatively large passive device between the supply rails of an integrated circuit is the most common technique to suppress power supply noise. Such devices, called decoupling capacitors, have been used both on- and off-chip for over 40 years. Their use enables improved noise immunity and consequently improve voltage regulation of the circuits. The metal-insulator-metal (MIM) capacitor is a capacitor widely used for decoupling purposes due to its high capacitance value while maintaining low parasitic capacitance and having low resistivity electrodes. In addition, MIM capacitors are able to deliver similar capacitance values to the multi-layer ceramic capacitors (MLCC) in much thinner layers. Therefore, MIM capacitors can be better integrated within the various levels of the chip fabrication process. As technology nodes advance to the nm regime, the existing MIM fabrication process needs to be further optimized in terms of capacitance density, reliability and space exploitation.

Capacitance densities may rise by reducing the dielectric thickness, which also achieves better device miniaturization for low voltage applications. However, this creates the problem of low breakdown voltage, poor voltage linearity and large leakage current. The introduction of dielectrics with higher dielectric constant than that of SiO_2_ (>3.9), so-called high-κ dielectrics, overcome some of these issues. High-κ materials exhibit a larger permittivity and band gap; thus, they enhance the capacitance while limiting the leakage simultaneously. In planar form, decoupling capacitors with high-κ dielectrics deposited by atomic layer deposition (ALD) at low temperatures (<650 K) have been investigated for competing against thick silicon oxide MIM capacitors [1]. Devices with high-κ ZrO_2_-based dielectric have already been reported for decoupling purposes [2]. ZrO_2_ has a wide band gap (5.1–7.8 eV) and a high dielectric constant, which varies from 17 to 47 depending strongly on its crystalline structure (monoclinic, cubic or tetragonal phase) [3,4].

To further increase the capacitance density of a MIM capacitor, while keeping the leakage current low, the shape of the capacitor has to change from a 2-dimensional to a 3-dimensional (3-D) structure. The increase in the surface-to-volume ratio for gaining higher capacitance densities has already been used in the past, for example in the case of DRAM cells with deep holes (trenches) etched in silicon or stacked oxide for miniaturizing the capacitors and increasing their storage [5]. Trenches with depth to width ratios (aspect ratios) of 50:1 are commonly used. It has been proven that the surface quality and the formation of the substrate in which the capacitor stack is deposited can strongly affect the final morphology of the capacitor [6], let alone in the case of 3-D structures where the density of the 3-D patterns as well as their geometry can further influence the electrical properties of the final product. Thus, conformal deposition and good uniformity of high-κ dielectrics inside high aspect ratio 3-D structures is challenging even in the case of ALD processing.

The influence of important ALD parameters on the electrical properties of the dielectric has been analyzed in previous studies in terms of the growth rate of the metal precursor or the variation in the oxidation pulse during the deposition of ZrO_2_-based 3-D MIM capacitors [7,8]. However, there are further ALD parameters, which affect the performance of the ALD process including the precursor pulse times and the deposition temperature which need to be understood to define the appropriate recipes for achieving a conformal deposition with low carbon content and proper sidewall coverage within the deep trenches of the 3-D MIM decoupling capacitors. Additionally, it has been shown that introducing Al with its large bandgap of 8.7 eV and its low dielectric constant of ~9 within the ZrO_2_-based high-κ stack keeps the dielectric amorphous during deposition, avoiding unwanted premature crystallization [2,8]. Consequently, high electric field strength, low oxygen diffusivity, lifetime improvement and lower leakage currents can be achieved [9,10]. Aluminum can be deposited with the same deposition technique (ALD) and it can be combined with the deposition of the high-k dielectric forming laminated or nano-laminated/mixed stacks. Specific capacitances of ~27 nF/mm^2^ have been reported for planar capacitors including nano-laminates of Al_2_O_3_ in ZrO_2_ [11].

The electrical data of the decoupling capacitors have to still fulfill the industrial specifications which dictate, for example, that the leakage current has to remain as low as possible (<1 µA/cm^2^) and the stationary capacitance over applied voltage (capacitance voltage linearity) has to remain consistent during the whole operating lifetime of the product to warrant the reliability of the capacitor. Moreover, long-term lifetime under various operating temperatures, regardless the size of the decoupling capacitor plays an essential role in all stages of product development. Temperature- and time-dependent dielectric breakdown (T-TDDB) should also meet industrial reliability requirements, which note that a minimum ten-year lifetime criterion at accumulated fail rate of 10 ppm should be satisfied. In this context, the appropriate process parameters, especially for the ALD process (Zr precursor pulse times, deposition temperature) of the Al_2_O_3_-doped ZrO_2_ high-dielectric stack have to be established, enabling optimum electrical properties.

In this work, we successfully fabricated 3-D MIM capacitors with an ALD deposited Al_2_O_3_-doped ZrO_2_ conformal and smooth high-κ dielectric stack with limited impurities on high aspect ratio substrates. We investigated the influence of the high-κ metal (Zr) precursor pulse time and the deposition temperature of the dielectric layers on the uniform coverage of the 3-D capacitors. The uniformity is a key performance factor as it is inextricably linked to the electric performance of the devices. We evaluated the fabricated devices experimentally with respect to electrical properties (capacitance and current dependence on voltage) and reliability parameters (TDDB).

## 2. Materials and Methods

**Figure 1:**
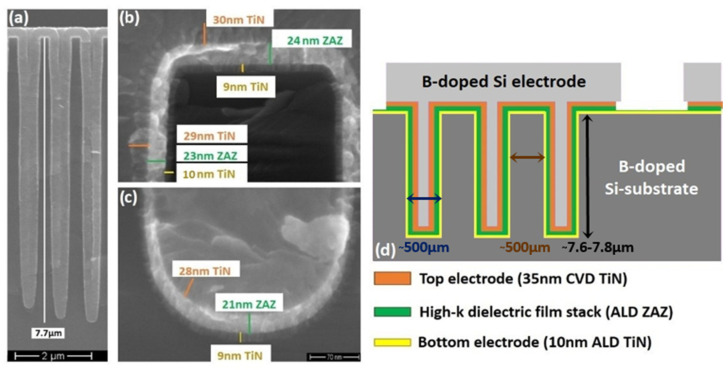
(**a**) SEM cross-section of a 7.7 µm deep array of trenches; (**b**) detail of one of the trenches in (**a**) at the top trench section; (**c**) detail of one of the trenches in (**a**) at the bottom of the trench; (**d**) schematic cross-section (not to scale) of the 3−D MIM capacitor realized in a silicon substrate. Bottom contact can be contacted on the front side or on the rear side.

The 3-D MIM capacitors (Figure 1a,d) were formed on highly boron-doped (0.02 Ω·cm), 775 µm thick, 300 mm silicon (100) wafers. In order to create the deep trenches needed for the 3-D shape of the capacitors, the wafers were structured using i-Line photolithography on a SiO_2_ hard-mask. Test devices with different footprint areas (0.01–0.1 mm^2^) and trench structures with depth of ~7.7 µm, as shown in Figure 1a, were defined and could be realized.

The wafers were then etched by the deep RIE process [12,13] and the openings/micro-trench arrays were formed. After finishing the structuring of the trenches by removing the hard-mask, a 10 nm thick TiN bottom electrode was deposited by ALD with TiCl_4_ and NH_3_ precursors at 723 K using an ASM A412 batch furnace.

**Figure 2:**
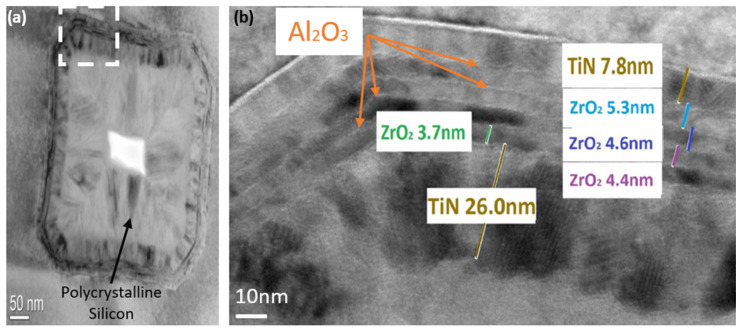
TEM micrographs of one individual trench of the deposited MIM stack with increased ALD temperature of 576 K and Zr precursor pulse times of 12 s/cycle: (**a**) bottom area of the 7.7 µm deep trench; (**b**) details of the sidewall of a trench with the MIM stack visible, where individual TiN electrode layers and the dielectric stack (ZrAl_x_O_y_) are indicated.

The high-κ dielectric films of Al_2_O_3_-doped ZrO_2_ (ZrAl_x_O_y_) with average thickness of ~23 nm (see in Figure 1b,c) and Figure 2b were deposited by atomic layer deposition (Jusung Eureka 3000, warm wall reaction chamber) using TEMAZr (Tetrakis-ethylmethylamino-zirconium, Zr[N(C_2_H_5_)CH_3_]_4_) and TMA (Tri-methyl-aluminum, Al(CH_3_)_3_) as metal precursors (353 K precursor bubbler temperature), ozone (O_3_) with flow rate of 200 g/m^3^ as reactant/oxidizing agent and Argon (Ar) as carrier/purge gas at deposition temperatures of 556 K or 576 K. The latter temperature has been reported to be the maximum allowed ALD temperature, before decomposition of TEMAZr begins [14]. The TEMAZr cycle consisted of pulsing with TEMAZr, purging with Ar gas, oxidizing with ozone, and purging with Ar gas. In a similar way, the dopant (TMA) was injected, followed by purging steps with Ar, O_3_ pulse and Ar purge again. In Table 1, the physical thickness values are presented which were measured on the top lateral surface of the high-κ film stack. TEMAZr pulse time was increased every time while decreasing the number of deposition cycles per super-cycle. Oxidation time (O_3_ pulse duration) followed the changes of the precursor pulse time.

After 48–61 cycles of TEMAZr and O_3_, ALD cycles of TMA with O_3_ followed, so that a thin film of Al_2_O_3_ was formed on top of the ZrO_2_ stack. The aluminum ALD cycles were changed following the variation in the TEMAZr pulse times in order to not affect the stoichiometry between Al and Zr in the dielectric stack. A series of ZrO_2_ cycles (48–61, depending on the pulse time of TEMAZr) followed by Al_2_O_3_ cycles defined a so-called super-cycle. By repeating this supercycle of ZrO_2_ and Al_2_O_3_ four times, the desired film thickness of the high-κ dielectric stack was achieved, with aluminum distributed across the dielectric stack. The last super-cycle consisted only of the TEMAZr cycle in order to have ZrO_2_ as the top layer of the high-k dielectric. There was no post deposition anneal (PDA) in order to keep the high-κ films in the amorphous state with a low defect density and high structural homogeneity [15]. As the top electrode, 35 nm of TiN were fabricated at 673 K by chemical vapor deposition (CVD). In Figure 2b, the slightly dark patches within ZrO_2_ with crystalline lattices indicate areas, partially crystallized into the tetragonal phase [16]. Based on Figure 2b, Al_2_O_3_ seemed to have helped not only during the deposition of the high-k stack at T_D_ = 556–576 K (Table 1) to avoid unwanted premature crystallization, but also during the remaining fabrication/integration process at temperatures up to 100 K higher than T_D_ to not fully crystalize the fabricated insulator.

**Table 1:** Experimental conditions and thickness values of Al_2_O_3_-doped ZrO_2_ dielectrics in MIM capacitors measured by spectral ellipsometry (Spectra FX 100, KLA Tencor).

TEMAZr Pulse Time (t_pulse_)	TEMAZr Cycles/Super-Cycle	Deposition Temperature (T_D_)	ZrAl_x_O_y_ Physical Thickness ^1^
3 s	61	283 °C/556 K	-
6 s	59	283 °C/556 K	24.6 nm
9 s	59	283 °C/556 K	28.7 nm
12 s	55	283 °C/556 K	29.1 nm
12 s	48	303 °C/576 K	27.4 nm
15 s	48	303 °C/576 K	27.0 nm

^1^ measured on the top lateral surface of the high-κ film stack.

A second photolithographic patterning step with a boron-doped silicon electrode was used to define the top electrode of the devices and served also as filling of the void of the trenches, while keeping the physical series resistance of the electrodes and therefore the Equivalent Series Resistance (ESR) as low as possible.

The morphology of the MIM capacitors were characterized by Scanning Electron Microscopy (S-5000, Hitachi). Samples were cleaved just before being loaded into the SEM chamber to maintain a fresh cross-sectional surface. Additionally, the MIM stack was visually analyzed using Transmission Electron Microscopy (F20 TEM 200 kV, FEI Tecnai) to support the SEM findings and additionally investigate more the high-κ dielectric formation in the trench structures. TEM was also used to investigate the crystal status of the fabricated samples after the end of the integration process.

**Figure 3:**
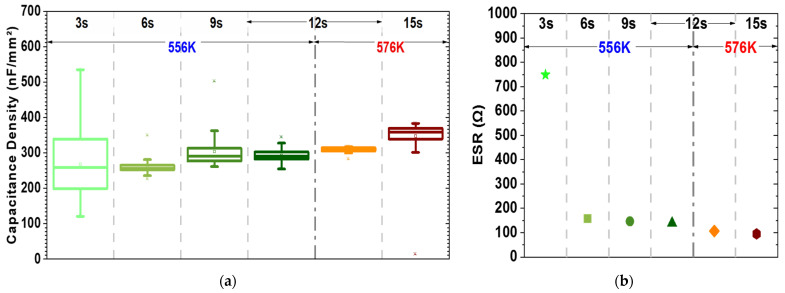
Variation over different TEMAZr pulse times and high-κ deposition temperatures measured at 0 V, 10 kHz and room temperature (298 K) for (**a**) capacitance density (C_0_) and (**b**) the median of equivalent series resistance (ESR).

The electrical characterization was performed on a temperature-controlled wafer stage of a fully automated semiconductor probe station (Precio Nano, Tokyo Electron) at room temperature (298 K). Capacitance (C-V) and leakage current (J-V) characteristics were determined using a C-V analyzer (E4980A, Agilent/Keysight) and a semiconductor parameter analyzer (B1500, Agilent/Keysight), respectively. In this work, a series equivalent circuit model was used for the capacitance measurements and voltage sweeps from negative to positive polarity directions were measured. As a way to have a high statistic over the wafer, all electrical measurements were executed on capacitors with a top lateral surface area of 0.01 mm^2^ and at minimum 53 dies per wafer. Throughout this work and more specifically in Figures 3a, 5a–c, 7a and 9, box-charts are used for the graphical representation of the key statistical values of the previously mentioned datasets of min. 53 capacitors. In particular, each separate set of data is represented as a separate box. The box is determined by the 25th and 75th percentiles. The whiskers are determined by the 5th and the 95th percentiles. The horizontal line in each box depicts the median and the open square symbol the mean, respectively. The samples were kept in the dark during electrical characterization. The area enhancement factor of the capacitors with high aspect-ratio holes compared to planar capacitors with similar surface area has been found to be ~20 in a previous experiment with the same setup and layout [17]. Within this work, the samples have always been normalized over the top lateral surface area of each capacitor.

## 3. Results and Discussion

### 3.1. Capacitance Behavior, Temperature Dependence and Voltage Linearity

Figure 3a depicts the results of the capacitance-voltage measurements at zero bias voltage (C_0_) and 10 kHz of the experiments of Table 1. The capacitance density values slightly increased with increasing TEMAZr pulse time. At 3 s pulse time and T_D_ = 556 K, the C_0_ reached ~260 nF/mm^2^ but with a very high deviation (±25%) over the wafer (non-uniformity). Due to the high non-uniformity and the high number of impurities, a parasitic parallel resistance appeared, increasing the equivalent series resistance (ESR) of the sample remarkably (~750 Ω), thus indicating a very high leakage (Figure 3b). By increasing the TEMAZr pulse time to 6 s and higher, the ALD process reached its saturation point, which can be verified by the significant lower deviation of the C_0_ values and the drop of the ESR values (Figure 3). Despite reaching the ALD saturation point, higher TEMAZr pulse times (9–12 s) were tested while aiming for a homogenous sidewall coverage all over the 3-D trench structure (bottom-middle-top). In such case, there was only a minimal increase in C_0_, which came together with slightly higher deviation of the values.

**Figure 4:**
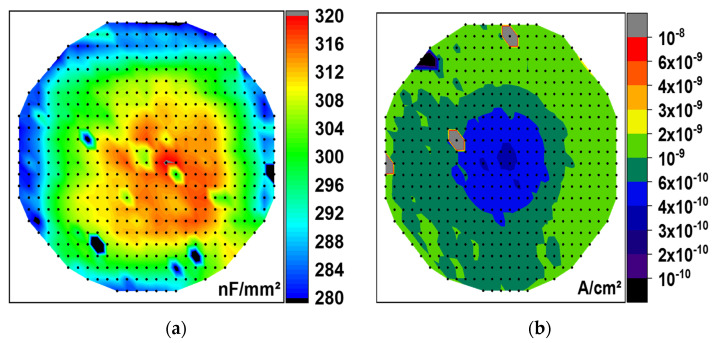
Wafer−maps of data measured at room temperature (298 K) on all (523) wafer−dies of a ZrAl_x_O_y_ dielectric stack with 12 s TEMAZr pulse time and 573 K deposition temperature: (**a**) C_0_ measured at frequency of 10 kHz; (**b**) leakage current density at +3 V plotted in logarithmic scale.

At the same time, the increase in the deposition temperature by 20 K (T_D_ = 576 K) while keeping the TEMAZr pulse time constant (12 s) improved the uniformity of C_0_ (see also Figure 4a) and slightly increased its value. A further increase of the pulse time to 15 s showed similar behavior to the elevated pulse times of 9 s and 12 s for T_D_ = 556 K, namely only a minimal rise in C_0_ with higher non-uniformity. The small thickness variations both inside the trench holes (Figure 2) as well as on the top surface of the capacitors have not significantly affected the capacitance behavior, but only improved the sidewall coverage.

**Figure 5:**
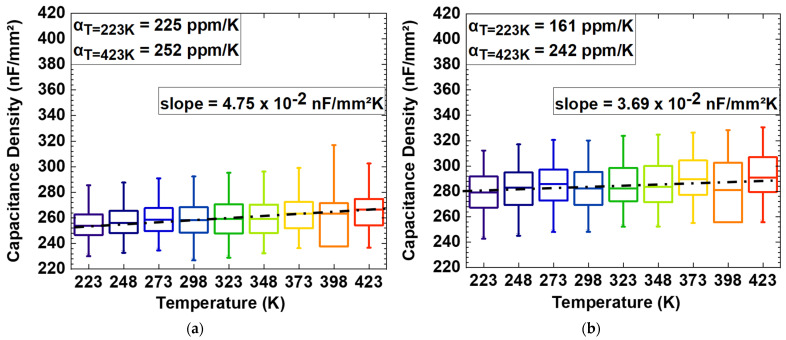

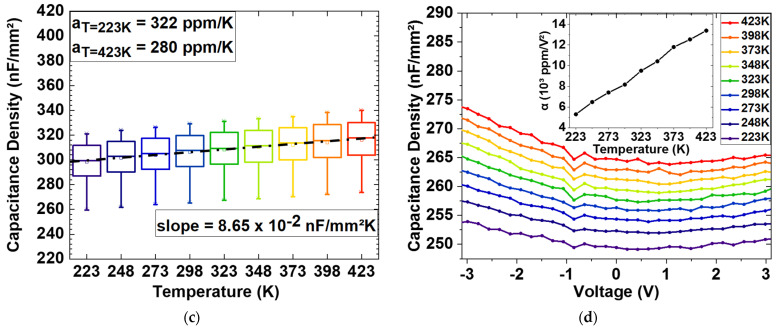
C_0_ over temperature (223 K up to 423 K), at measurement frequency of 10 kHz for: (**a**) 6 s TEMAZr pulse time and T_D_ = 556 K, (**b**) 12 s TEMAZr pulse time and T_D_ = 556 K deposition temperature and (**c**) 12 s TEMAZr pulse time and T_D_ = 576 K; (**d**) C(V)−plot over temperature of the ZrAl_x_O_y_ stack of (**c**).

The dependence of the capacitance density on temperature for increasing TEMAZr pulse time and deposition temperature was studied under a wide temperature range (223–423 K), as depicted in Figure 5a–c. From these outcomes, the performance of capacitance can be checked in extreme operating environmental conditions, similarly to those expected for automotive applications [18]. In all cases, there is a temperature acceleration and the capacitance density at zero bias (C_0_) was found to vary in a fairly linear manner with respect to temperature. By increasing the TEMAZr pulse time from 6 s to 12 s, negligible changes appear in the C_0_/T slope (~4.2 nF/mm^2^ K). On the other hand, by increasing the deposition temperature from 556 K to 576 K, the temperature acceleration rate doubles (~8.7 nF/mm^2^ K). The majority of the defects seem to be caused during the ozone steps at higher deposition temperature. These defects are activated by temperature and start acting as traps. By that, more charges can reach the electrodes faster while the rate of applied voltage remains constant. Thus, the capacitance density increases based on the standard equation of dq/dt=CdV/dt.

The temperature coefficient of capacitance $\alpha_{T}$, a figure of merit of the dielectric capacitor devices, which describes the maximum change in capacitance density over a specified temperature range, is formulated as

(1) $$\alpha_{T}=\frac{\frac{C_{T}}{C_{\mathrm{RT}}}-1}{T\;-{\;T}_{\mathrm{RT}}}\cdot 10^{6}\mathrm{ppm}/K$$

where C_T_ and C_RT_ are the capacitance values at a particular temperature limit T and at room temperature (T_RT_ = 298 K), respectively. Room temperature is established as a reference temperature. The numerical values for 12 s TEMAZr pulse time and T_D_ = 576 K were determined to be 322 ppm/K and 280 ppm/K at the extreme temperatures of 223 and 423 K, respectively and are within the limit of 750 ppm/K set by the Electronic Industries Alliance (EIA) for capacitors [19].

An important parameter of a MIM capacitor for decoupling applications is its capacitance stability over bias voltage—also called voltage linearity. The voltage linearity of MIM capacitors is determined by a second-order polynomial equation

(2) $$\frac{C\left(V\right)}{C_{0}}-1={\alpha V}^{2}+\beta V$$

where V is the applied bias voltage; C(V), the capacitance density at a particular bias voltage; C_0_ the zero-bias capacitance; α and β, the quadratic and linear Voltage Capacitor Coefficients (VCC), expressed in units of parts per million (ppm)/V^2^ and ppm/V, respectively.

The capacitance density values for the ZrAl_x_O_y_ stack with TEMAZr pulse time of 12 s and T_D_ = 576 K were measured at a measurement frequency of 10 kHz from −3 V to +3 V with a voltage step of +0.2 V at temperatures from 223 K to 423 K and are depicted in Figure 5d. The data was fitted with the parabolic curve of Equation (2). The extracted quadratic term α exhibits a linear relationship with respect to the applied temperature (see the inset of Figure 5d) which can be expected from the electrode polarization model and explained by thermally enhanced mobility of charge carriers in the dielectric [6].

Both the temperature coefficient of capacitance $\alpha_{T}$ (1) and the voltage capacitance coefficients, α and β (2) are independent of the area enhancement factor of the 3-D capacitors.

**Figure 6:**
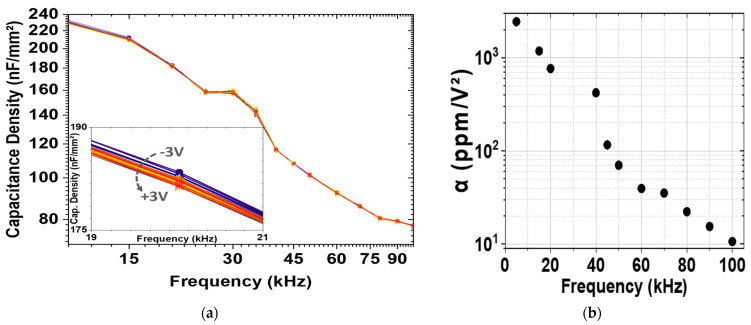
(**a**) Capacitance density values for the high−κ stack with 12 s Zr pulse time and 576 K deposition temperature measured at room temperature (298 K) with applied voltages from −3 V to +3 V and measurement frequencies from 5 kHz to 100 kHz. (Inset: zoomed part of the C−f plot, where the different bias voltages at 20 kHz are shown); (**b**) behavior of the quadratic VCC α over the measurement frequencies.

Another important parameter, which defines the quality of the decoupling capacitors, is their performance over different applied frequencies. Figure 6 shows the plot of capacitance density values measured from −3 V to +3 V at different frequencies (10–100 kHz) for 12 s TEMAZr pulse time and T_D_ = 576 K. On the one hand, the capacitance density remained nearly stable over voltage at any fixed applied frequency and voltage polarity, which indicates the good capacitor’s stability under a continuously increasing voltage stress. On the other hand, by increasing the measurement frequency, the capacitance was notably reduced. Because of the increasing measurement frequencies, the polarization density $P\left(t\right)=P_{0}+\int_{0}^{t}J_{P}\left(t\right)dt$ cannot fully respond due to dielectric relaxation. Consequently, the capacitance density drops, regardless the amount of the applied bias voltage. In the case of 3-D decoupling capacitors with high aspect ratio structures, this behavior becomes more noticeable due to their complex morphologies, such as edges, corners, and sidewall spikes, resulting from the etching process. These cause the localization of enhanced electric field which might further delay the response of charge and thus cause a reduction in the quadratic VCC, as depicted in Figure 6b, where the extracted α values decreased from 2300 ppm/V^2^ to 10 ppm/V^2^ with increasing frequency.

### 3.2. Leakage Currents, Breakdown and Reliability Characteristics

**Figure 7:**
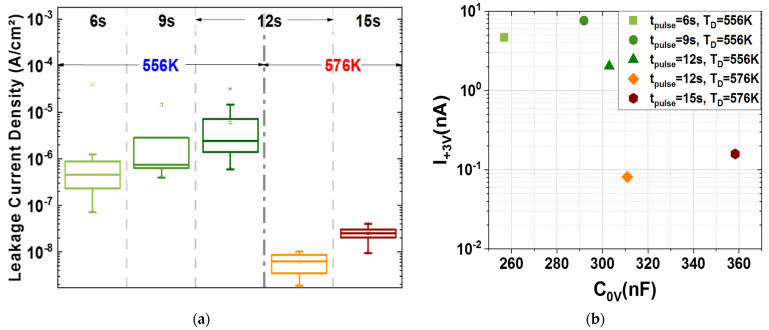
(**a**) Leakage current density measured at +3 V bias voltage for different TEMAZr pulse times and high−κ deposition temperatures; (**b**) Figure of Merit plot showing the behavior of the capacitor as a relation between the leakage current at +3 V bias voltage (2.5 times higher than the operational voltage) and the capacitance without bias voltage; median values of I_+3V_ and C_0V_ are presented for each ALD recipe in this plot. In both plots, (**a**,**b**), the samples were measured at room temperature (298 K).

The leakage current density of the 3-D MIM capacitors is a critical factor in terms of reliability, especially for the very thin embedded MIM capacitors. The plot in Figure 7a reveals very low values (<1 µA/cm^2^) for all tested material stacks at room temperature (298 K) and +3 V bias voltage. With increasing the deposition temperature (T_D_ = 576 K) of the high-κ dielectric stack, the uniformity of the leakage current data increased. The increase in the deposition temperature together with a higher TEMAZr pulse time proved to be the most beneficial as it allowed capacitors not only to have an optimal low leakage current, but also the overall highest capacitance, which can be extracted from the I/C-plot in Figure 7b. Additionally to the improved uniformity of the leakage current over the wafer (Figure 4b), samples obtained with equal TEMAZr pulse time (12 s) but higher T_D_, demonstrated absolute values of leakage current density, which were decreased by almost two orders of magnitude. This large reduction can be related to both lower carbon impurities during the ALD deposition process and the lightly better step coverage at higher temperatures.

**Figure 8:**
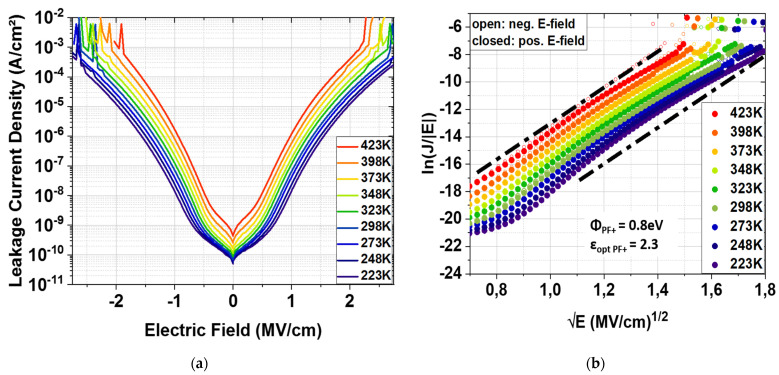
(**a**) Leakage current density over the electric field at measuring temperatures from 223 K to 423 K; (**b**) Poole−Frenkel coordinates over temperature for both field polarities. Both data refer to the samples with 12 s TEMAZr pulse time and T_D_ = 556 K.

Additionally, the leakage current behavior of the sample with the lowest leakage has been characterized under temperature for both voltage polarities (Figure 8a). An almost perfect symmetry of the leakage current density curves for both polarities could be extracted. The leakage current raised with increasing measurement temperatures. This increase can be related to the thermally enhanced mobility of charge carriers. It has been reported before [6] that in case of amorphous Al-doped ZrO_2_ high-κ thin films with a dielectric thickness higher than 10 nm, Poole-Frenkel electron emission from traps in the dielectric is the dominant conduction mechanism due to the significant temperature dependence of the J-E characteristics. Additionally, the symmetry of the leakage current density curves for both polarities is a strong indicator both for non-fully crystalline dielectric and for Poole-Frenkel emission in case of MIM capacitors using metals with different work functions [20,21]. The Poole–Frenkel effect describes the way electrons in insulators use the thermal fluctuations to be promoted to the conduction band to travel between traps. The applied field lowers the barrier of the trap; hence a smaller work function is needed to overcome it [22]. Having that in mind and to further understand the conduction mechanism of 3-D MIM decoupling capacitors at low and high electric field strengths in both polarities, the J-E plot was re-plotted in Poole-Frenkel coordinates (Figure 8b) and the values were fitted with the Poole-Frenkel (PF) acceptor limiting conduction current:

(3) $$J_{\mathrm{PF}}\propto {\mathrm{Ee}}^{\frac{-q\left(\varphi_{\mathrm{PF}}-\sqrt{\mathrm{qE}/{\pi \varepsilon }_{0}\varepsilon_{\mathrm{opt}}}\right)}{k_{B}T}}$$

where φ_PF_ describes the trap depth without external E-field, ε_opt_ denotes the optical dielectric constant, q is the electron-charge, E stands for the electrical field applied to the film, k_B_ is the Boltzmann’s constant and T is the applied temperature [21]. Τhe PF-plot can be well-fitted in both polarities by a straight line up to very low field values of high electric field region, which is in line with what was previously published [6].

**Figure 9:**
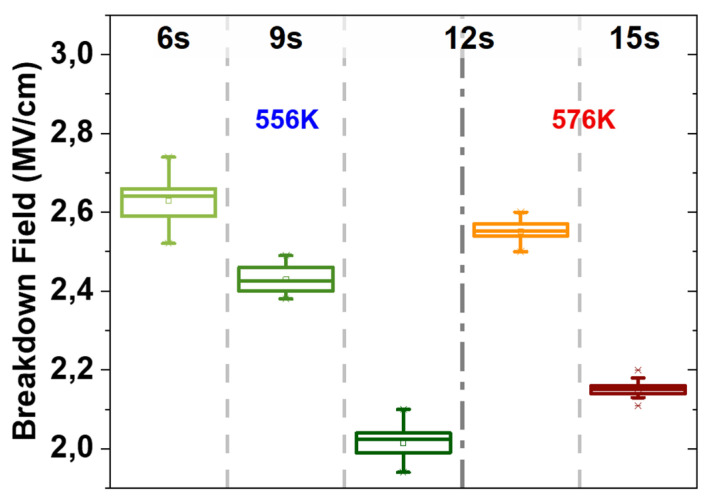
Breakdown Field at room temperature for increasing TEMAZr pulse time and deposition temperatures.

After measuring the leakage current density within the operating voltage range of the MIM decoupling capacitors (±3 V), breakdown voltage values and Time Dependent Dielectric Breakdown (TDDB) were also measured at temperatures from 223 K to 423 K on wafer level, using a voltage ramp stress (VRS) and constant voltage stress (CVS), respectively. To enable the comparison of different stacks/ALD parameters with different thickness values, the breakdown electric field strength was used, calculated according to the standard equation of E = V/d, assuming there is no electron shielding due to the absence of ferroelectricity [23]. Measurement of breakdown for 523 dies (full map) yielded values between 2.05 MV/cm and 2.65 MV/cm (Figure 9) with no early breakdown registered. This indicates a low defect density because of the good quality of the deposition on the deep-trench structures. By increasing TEMAZr pulse times, similarly to the behavior of the leakage current density, more impurities caused lower breakdown fields despite the better trench-sidewall coverage. As an example, 6 s higher pulse time caused a drop of the breakdown field by 30%. Higher T_D_ increased the breakdown of the films again to higher values.

The trade-off between the capacitance, the leakage current and the breakdown field, the standard electrical characterization parameters of a MIM capacitor, as depicted in Figure 7b and Figure 9 indicated that the best ALD recipe proved to be the one with 12 s TEMAZr pulse time and higher T_D_.

The high breakdown field strength generally enables capacitors to have a large and stable working voltage and reflects the capacitor’s lifetime. In order to analyze the reliability of the 3-D MIM decoupling capacitors, the stress time required for dielectric breakdown under constant field stress was measured which is also called TDDB lifetime. For the TDDB measurements, the capacitors are subject to field stress in a range of 1.8–2.6 MV/cm. For each field stress value, multiple capacitors, each on an individual die, are assessed according to previously measured breakdown voltage and breakdown current density characteristics. The time to breakdown (t_BD_) is measured individually with a sampling interval of 100 ms and a current compliance of 1 mA. From the measured t_BD_ data the breakdown time at 63.2% failure of the devices (t_63.2_) is extracted, which for the purpose of this paper will be called characteristic lifetime. The characteristic lifetime data is then extrapolated to the operating electric field by fitting the data to the “linear E” model and calculating the voltage acceleration γ. This model was selected because it describes the metal-oxide bond breakage [24] while also being a more conservative model compared to the power law model used before for similar devices [25].

**Figure 10:**
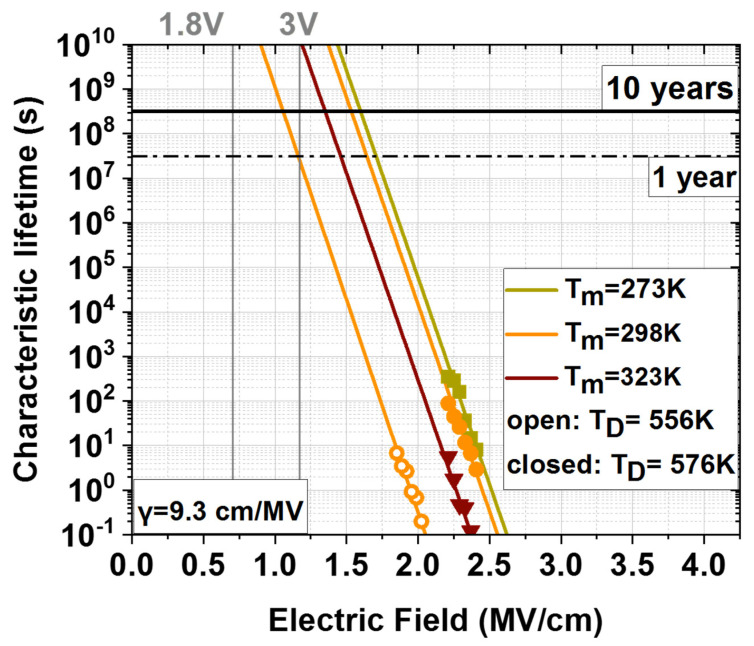
Characteristic lifetime of MIM devices with 12 s TEMAZr pulse time at both deposition temperatures (553 K, 573 K) for different stress fields measured at 273 K, 298 K and 323 K. Lifetime extrapolated to 10 years of lifetime using an exponential relation with voltage acceleration γ being the slope.

As shown in Figure 10, the extrapolation of the characteristic lifetime to the operating electric field (voltage) of ~1.2 MV/cm (3 V) yielded average values of >10^10^ s or more than 1000 years for samples with 12 s Zr precursor pulse time and an ALD deposition temperature of 576 K. This performance was confirmed for all measuring temperatures (T_m_) up to 323 K. The characteristic lifetime of the 3-D MIM decoupling capacitors under room temperature conditions (298 K) has been improved by almost two orders of magnitude by increasing T_D_ by 20 K due to the reduction of impurities.

## 4. Conclusions

Embedded 3-D MIM decoupling capacitors with Al_2_O_3_-doped ZrO_2_ high-κ dielectric film stack have been realized on silicon wafers. The behavior of the samples with respect to the complex morphologies of the 3-D trenches and ALD deposition conditions has been analyzed. Their electrical and reliability properties were investigated by capacitance, leakage current, dielectric breakdown and TDDB measurements. We have shown that with increased ALD deposition temperature (576 K) and longer Zr precursor pulse times (12 s/cycle) close to the precursor saturation point, the capacitors revealed a very high uniformity (>90%). Additionally, capacitance density with average values of 350 nF/mm^2^ and low leakage current of <1 µA/cm^2^ at +1.1 MV/cm (~+3 V) were measured. In addition, the leakage current behavior over temperature has been theoretically examined and can be attributed to the Poole-Frenkel effect. The breakdown field reached a maximum of ±2.7 MV/cm (±8 V). Finally, a characteristic lifetime of more than 10 years at the operating voltage of 3 V could be achieved successfully. These results, which meet the performance standards required from the semiconductor industry, highlight the importance of the careful tuning of the metal precursor and the ALD deposition temperature in high-κ dielectric stack systems used for 3-D decoupling MIM capacitors. Moreover, they emphasize the need for the further understanding of the interactions of ALD processes for such thin ZrAl_x_O_y_ films. With that understanding, the material system can be further improved and become a more reliable candidate for embedded decoupling 3-D MIM capacitors.

## Data Availability

Research data in this article is not shared.

## References

[1] Jenkins M., Austin D., Conley J., Fan J., de Groot C.H., Jiang L., Fan Y., Ali R., Ghosh G., Orlowski M. (2019). Review—Beyond the Highs and Lows: A Perspective on the Future of Dielectrics Research for Nanoelectronic Devices. ECS J. Solid State Sci. Technol..

[2] Seidel K., Weinreich W., Polakowski P., Triyoso D.H., Nolan M.G., Yiang K.Y., Chu S. Reliability comparison of pure ZrO_2_ and Al−doped ZrO_2_ MIM capacitors. Proceedings of the 2013 IEEE International Integrated Reliability Workshop Final Report.

[3] Lee J.-H., Park B.-E., Thompson D., Choe M., Lee Z., Oh I.-K., Kim W.-H., Kim H. (2020). Improved interface quality of atomic-layer-deposited ZrO2 metal-insulator-metal capacitors with Ru bottom electrodes. Thin Solid Films.

[4] Ferrand J., Beugin V., Crisci A., Coindeau S., Jeannot S., Gros-Jean M., Blanquet E. (2013). Tetragonal Zirconia Stabilization by Metal Addition for Metal-Insulator-Metal Capacitor Applications. ECS Trans..

[5] Amon J., Kieslich A., Heineck L., Schuster T., Faul J., Luetzen J., Fan C., Huang C.C., Fischer B., Enders G. A highly manufacturable deep trench based DRAM cell layout with a planar array device in a 70nm technology. Proceedings of the IEDM Technical Digest. IEEE International Electron Devices Meeting.

[6] Weinreich W., Wilde L., Müller J., Sundqvist J., Erben E., Heitmanm J., Lemberger M., Bauer A.J. (2013). Structural Properties of as deposited and annealed ZrO_2_ influenced by atomic layer deposition, substrate and doping. J. Vac. Sci. Technol. A.

[7] Paskaleva A., Weinreich W., Bauer A.J., Lemberger M., Frey L. (2015). Improved electrical behavior of ZrO2-based MIM structures by optimizing the O_3_ oxidation pulse time. Mater. Sci. Semicond. Process..

[8] Weinreich W., Tauchnitz T., Polakowski P., Drescher M., Riedel S., Sundqvist J., Seidel K. (2013). TEMAZ/O_3_ atomic layer deposition process with doubled growth rate and optimized interface properties in metal–insulator–metal capacitors. J. Vac. Sci. Technol. A.

[9] Wilk G.D., Wallace R.M., Anthony J.M. (2001). High-κ gate dielectrics: Current status and materials properties considerations. J. Appl. Phys..

[10] Schröder U., Weinreich W., Erben E., Müller J., Wilde L., Heitmann J., Agaiby R., Zhou D., Jegert G., Kersch A. (2009). Detailed Correlation of Electrical and Breakdown Characteristics to the Structural Properties of ALD Grown HfO_2_- and ZrO_2_-based Capacitor Dielectrics. ECS Trans..

[11] Mart C., Zybell S., Riedel S., Czernohorsky M., Seidel K., Weinreich W. (2017). Enhanced reliability and capacitance stability of ZrO_2_ –based decoupling capacitors by interface doping with Al_2_O_3_. Microelectron. Eng..

[12] Engelhardt M. (1991). Single-crystal silicon trench etching for fabrication of highly integrated circuits. Advanced Techniques for Integrated Circuit Processing.

[13] Adler E., DeBrosse J.K., Geissler S.F., Holmes S.J., Jaffe M.D., Johnson J.B., Koburger C.W., Lasky J.B., Lloyd B., Miles G.L. (1995). The evolution of IBM CMOS DRAM technology. IBM J. Res. Dev..

[14] Hausmann D.M., Kim E., Becker J., Gordon R.G. (2002). ALD of Hafnium and Zirconium Oxides using metal amide precursors. Chem. Mater..

[15] Zhang Q. (2019). The effects of Annealing on the Electrical Performance of MIM Capacitors with ZrO_2_ Dielectric. Integr. Ferroelectr..

[16] Weinreich W., Seidel K., Rudolph M., Koch J., Paul J., Riedel S., Sundqvist J., Steidel K., Gutsch M., Beyer V. Scaling and optimization of high-density integrated Si-capacitors. Proceedings of the 2013 International Semiconductor Conference Dresden—Grenoble (ISCDG).

[17] Kühnel K., Czernohorsky M., Mart C., Weinreich W. (2019). High-density energy storage in Si-doped hafnium oxide thin films on area-enhanced substrates. J. Vac. Sci. Technol. B.

[18] AEC Component Technical Committee (2014). Failure Mechanism Based Stress Test Qualification For Integrated Circuits. AEC-Q100, Rev-H, Automotive Electronics Council, USA. http://www.aecouncil.com/Documents/AEC_Q100_Rev_H_Base_Document.pdf.

[19] (2002). Electronic Components, Assemblies & Materials Association Ceramic Dielectric Capacitors Classes I, II, III and IV—Part I: Characteristics and Requirements. EIA-198-1, Rev-F, Electronic Industries Alliance, Arlington, USA. https://www.antpedia.com/standard/pdf/129263/1804/1524013638-4568.pdf.

[20] Weinreich W., Reiche R., Lemberger M., Jegert G., Müller J., Wilde L., Teichert S., Heitmann J., Erben E., Oberbeck L. (2009). Impact of interface variations on J-V and C-V polarity asymmetry of MIM capacitors with amorphous and crystalline Zr_(1- x)_Al_x_O_2_ films. Microelectron. Eng..

[21] Yeargan J.R., Taylor H.L. (1968). The Poole-Frenkel Effect with Compensation Present. J. Appl. Phys..

[22] Ongaro R., Pillonnet A. (1989). Poole-Frenkel (PF) effect high field saturation. Rev. Phys. Appliquée Société Française Phys./EDP.

[23] Brennan C.J. (1992). Characterization and modelling of thin-film ferroelectric capacitors using C-V analysis. Integr. Ferroelectr. Int. J..

[24] Strand J., La Torraca P., Padovani A., Larcher L., Shluger A.L. (2002). Dielectric breakdown in HfO_2_ dielectrics: Using multiscale modeling to identify the critical physical processes involved in oxide degradation. J. Appl. Phys..

[25] Zhou D., Schroeder U., Xu J., Heitmann J., Jegert G., Weinreich W., Kerber M., Knebel S., Erben E., Mikolajick T. (2010). Reliability of Al_2_O_3_-doped ZrO_2_ high-k dielectrics in three-dimensional stacked metal-insulator-metal capacitors. J. Appl. Phys..

